# Editorial: Immunotherapy for Prostate Cancer – turning the immunological desert into an oasis of hope

**DOI:** 10.3389/fonc.2022.1021870

**Published:** 2022-09-08

**Authors:** Christine Galustian, Angus Dalgleish, Mark Bodman-Smith, Sergei Kusmartsev, Prokar Dasgupta

**Affiliations:** ^1^ Peter Gorer Department of Immunobiology, School of Immunology and Microbial Sciences, King’s College London, London, United Kingdom; ^2^ Institute for Infection and Immunology, St. George’s, University of London, London, United Kingdom; ^3^ Department of Urology, School of Medicine, University of Florida, Gainesville, FL, United States

**Keywords:** Prostate Cancer, Immunotherapy, Tumor microenvironment, Castration-resistant Prostate Cancer, CAR T cell therapy, Provenge^®^

Globally, prostate cancer is the second-most common cancer. It is the fifth-leading cause of cancer-related death in men. It was the most common cancer in males in 84 countries, occurring more commonly in the developed world. Treatment for prostate cancer may involve surgery, radiation therapy, hormonal therapy, chemotherapy, or some combination therapy. Treatments also extend to survivorship-based interventions. However, despite many recent advances in the therapy for metastatic castration-resistant prostate cancer (mCRPC), the disease remains incurable.

Immunotherapy is one of the promising avenues for the future treatment of prostate cancer including mCRPC. The goal of most approaches to cancer immunotherapy is to activate a population of effector T lymphocytes, which can upon recruitment to the tumor exert the specific lysis of malignant cells. However, in recent years it has been shown that immunotherapy of prostate cancer is not very effective for several reasons. First, prostate cancer has been described as an immunological desert, where few T cells infiltrate tumors, compared to more immunogenic cancers such as melanoma. The prostate tumor microenvironment (TME) is highly immunosuppressive: The stromal fibroblasts and macrophages interacting with the cancer epithelial cells create a milieu that renders activated infiltrating T cells and NK cells anergic or regulatory1 (1, 2). In addition, the numbers of neoantigen-specific T cells created are much less than those seen in melanoma as Prostate cancer typically has a low tumor mutational burden (TMB) (5-10% of the mutational base pairs observed in Melanoma), which decreases the numbers and repertoire of neoantigens created (3). Consequently, the vast majority of prostate cancer patients do not respond to checkpoint inhibitors (anti-CTLA-4, anti-PD1, or anti-PD-L1) (4). There are also challenges with the use of agents directed to single checkpoints as tumors may express many checkpoints. In prostate cancer, several immune checkpoints have been observed, including B7H3 (CD276), LAG-3 (Lymphocyte Activating 3), TIGIT (T cell immunoreceptor with Ig and ITIM domains), and VISTA (V-domain Ig suppressor of T cell activation) (5). VISTA, for example, is shown to be upregulated after Ipilimumab (Anti-CTLA-4) therapy in patients with prostate cancer (6).

Sipuleucel-T (Provenge™) is currently the only form of immunotherapy approved for the treatment of mCRPC, and the first therapeutic cancer vaccine for prostate cancer to be approved by the U.S. Food and Drug administration (FDA). Provenge™ is an autologous dendritic cell vaccine prepared by taking a patient’s peripheral blood mononuclear cells, including antigen-presenting cells, initially extracted by leukapheresis and then treated with a fusion protein consisting of Granulocyte-macrophage colony-stimulating factor (GMCSF) and the antigen prostate acid phosphatase (PAP) associated with prostate tumors. These cells, once injected back into the patients can then present antigen to prime T cells into becoming PAP antigen-specific cytotoxic T cells directed against the patients’ tumors (7–9). The vaccine extends life by an average of four months.

The second promising approach is radioimmunotherapy. Developments in radioimmunoconjugates are likely to further transform the treatment of advanced prostate cancer, for example, with antibodies to prostate-specific membrane antigen (PSMA) linked to alpha particles emitting radioisotopes showing significant potential for patient benefit.

Another highly promising modality for the treatment of prostate cancer is CAR T-cell therapy. Chimeric antigen receptors (CAR) are synthetic receptors that redirect T cells to target cancer cells in a major histocompatibility complex–independent manner. CAR T-cell therapy has shown remarkable and unparalleled responses in patients with refractory or relapsed acute lymphocytic leukemia. However, despite the tremendous impact of CD19-directed CAR T-cell therapy, primary resistance or relapse is frequently observed in patients with types of malignancies. It has become evident that the efficacy of CAR T-cell therapies targeting solid tumors is limited due to the immunosuppressive nature of the tumor microenvironment in cancers such as prostate cancer (10). The tumor-associated immune suppression greatly affects the quantity, persistence, and functionality of T cells transferred to the tumor host.

Indeed, both immunosuppressive tumor microenvironment and resistance to immunotherapy remain major obstacles to the development of successful novel immunotherapies for prostate cancer. Despite the aforementioned knowledge, the mechanisms of resistance are unclear and need further elucidation. This Research Topic, therefore, provides a great opportunity to highlight and promote research in this area. It is a concise collection of preclinical and clinical studies based on recent findings in prostate cancer research.

Research by Zuccolotto et al is focused on PSMA-specific CAR-engineered T Cells for the treatment of prostate cancer. It shows that the second-generation CAR construct based on CD3-CD28 signaling outperformed the CAR T cells engineered using the third-generation CD3-CD28-41BB link. The authors suggest that strong signals induced in 3rd generation CAR T cells may lead to early cell exhaustion and activation-induced cell death (AICD). Therefore, this study also shows the importance of optimizing CAR construction and highlights the relevance to understand how receptor structure acts on transgenic T cell functionality, especially now that it is clear that some CARs cause exhaustion and impaired T cell activity.

The study by Zhao et al uses bioinformatics to identify novel targets for Prostate cancer therapy: Using a large number of datasets obtained with bioinformatics methods and cell experiments they studied changes in the prostate cancer microenvironment and identified the core genes that affect the microenvironment. 591 samples were collected from the Cancer Genome Atlas (TCGA) and Gene Expression Omnibus (GEO) cohorts and evaluated for the abundance and distribution of immune cell members in the prostate cancer microenvironment. The authors found that prostate cancer tissues are enriched in genes that mediate the immunosuppressive JAK/STAT3 pathway. They also report that the mechanism underlying such senescence induction may be potentially mediated via down-regulation of lactoferrin (LTF) in the prostate tumor microenvironment.

Another bioinformatics study by Ju et al provides a basic machine-learning computational framework for the signature of immunosuppressive T regulatory cells (T regs) in prostate cancer. The authors identified a T reg-specific prognostic signature (TILTregsSig) among tumor-infiltrating lymphocytes, which displays an independently predictive potential for the prognosis of prostate cancer patients.

The study by Feng et al employs a wide variety of techniques to identify a novel monogram to predict prostate cancer progression based on immune infiltrate and the circadian clock. They identified a gene signature to predict progression probability and found that patients prone to prostate cancer progression express several genes including PTGS2 (encoding prostaglandin-endoperoxide synthase 2, or COX2), CDKN3 (cyclin-dependent kinase inhibitor-3), SLC25A27 (Mitochondrial uncoupling protein 4), GUCY2C (Guanylate cyclase 2C). The authors also demonstrate that tumor samples have significantly higher infiltration levels of myeloid cells including macrophages and dendritic cell subsets.

The study by Xu et al. underscores the roles of m5C RNA modification patterns in biochemical recurrence and tumor microenvironment in prostate adenocarcinoma. They found that TET2, which was highly expressed in adjacent normal tissues compared to tumor tissues, was closely associated with many infiltrating immune cells. The m5C modification signature was constructed for the potential clinical application. The risk score calculated by the m5C signature was associated with the T stage, N stage, and Gleason score.

Overall, the knowledge offered in these articles is beneficial to building a clearer picture of the immunotherapy of prostate cancer. However, much more extensive investigations on this Research Topic are still required for developing new immunotherapeutic approaches, and further enhancing our understanding of the mechanisms of therapeutic resistance with an ultimate goal to boost therapeutic efficacy and improve the clinical outcome.

## Author contributions

All authors listed have made a substantial, direct, and intellectual contribution to the work, and approved it for publication.

## Acknowledgments

We would like to thank all authors for their contribution to our Research Topic.

## Conflict of interest

SK is a founder of K-Lab Therapeutics.

The remaining authors declare that the research was conducted in the absence of any commercial or financial relationships that could be construed as a potential conflict of interest.

## Publisher’s note

All claims expressed in this article are solely those of the authors and do not necessarily represent those of their affiliated organizations, or those of the publisher, the editors and the reviewers. Any product that may be evaluated in this article, or claim that may be made by its manufacturer, is not guaranteed or endorsed by the publisher.
